# Alcohol Consumption Patterns and Mortality Among Older Adults With Health-Related or Socioeconomic Risk Factors

**DOI:** 10.1001/jamanetworkopen.2024.24495

**Published:** 2024-08-12

**Authors:** Rosario Ortolá, Mercedes Sotos-Prieto, Esther García-Esquinas, Iñaki Galán, Fernando Rodríguez-Artalejo

**Affiliations:** 1 Department of Preventive Medicine and Public Health, Universidad Autónoma de Madrid, Madrid, Spain; 2 Center for Biomedical Research in Epidemiology and Public Health, Madrid, Spain; 3 Department of Environmental Health and Nutrition, Harvard T.H. Chan School of Public Health. Boston, Massachusetts; 4 Madrid Institute for Advanced Studies Food Institute, Campus of International Excellence Universidad Autónoma de Madrid + Spanish National Research Council, Madrid, Spain; 5 Department of Chronic Diseases, National Center for Epidemiology, Carlos III Health Institute, Madrid, Spain

## Abstract

**Importance:**

Alcohol consumption is a leading cause of morbidity and mortality that may be more important in older adults with socioeconomic or health-related risk factors.

**Objective:**

To examine the association of alcohol consumption patterns with 12-year mortality and its modification by health-related or socioeconomic risk factors.

**Design, Setting, and Participants:**

This prospective cohort study used data from the UK Biobank, a population-based cohort. Participants were current drinkers aged 60 years or older. Data were analyzed from September 2023 to May 2024.

**Exposure:**

According to their mean alcohol intake in grams per day, participants’ drinking patterns were classified as occasional: ≤2.86 g/d), low risk (men: >2.86-20.00 g/d; women: >2.86-10.00 g/d), moderate risk (men: >20.00-40.00 g/d; women: >10.00-20.00 g/d) and high risk (men: >40.00 g/d; women: >20.00 g/d).

**Main Outcomes and Measures:**

Health-related risk factors were assessed with the frailty index, and socioeconomic risk factors were assessed with the Townsend deprivation index. All-cause and cause-specific mortality were obtained from death certificates held by the national registries. Analyses excluded deaths in the first 2 years of follow-up and adjusted for potential confounders, including drinking patterns and preferences.

**Results:**

A total of 135 103 participants (median [IQR] age, 64.0 [62.0-67.0] years; 67 693 [50.1%] women) were included. In the total analytical sample, compared with occasional drinking, high-risk drinking was associated with higher all-cause (hazard ratio [HR], 1.33; 95% CI, 1.24-1.42), cancer (HR, 1.39; 95% CI, 1.26-1.53), and cardiovascular (HR, 1.21; 95% CI, 1.04-1.41) mortality; moderate-risk drinking was associated with higher all-cause (HR, 1.10; 95% CI, 1.03-1.18) and cancer (HR, 1.15; 95% CI, 1.05-1.27) mortality, and low-risk drinking was associated with higher cancer mortality (HR, 1.11; 95% CI, 1.01-1.22). While no associations were found for low- or moderate-risk drinking patterns vs occasional drinking among individuals without socioeconomic or health-related risk factors, low-risk drinking was associated with higher cancer mortality (HR, 1.15; 95% CI, 1.01-1.30) and moderate-risk drinking with higher all-cause (HR, 1.10; 95% CI, 1.01-1.19) and cancer (HR, 1.19; 95% CI, 1.05-1.35) mortality among those with health-related risk factors; low-risk and moderate-risk drinking patterns were associated with higher mortality from all causes (low risk: HR, 1.14; 95% CI, 1.01-1.28; moderate risk: HR, 1.17; 95% CI, 1.03-1.32) and cancer (low risk: HR, 1.25; 95% CI, 1.04-1.50; moderate risk: HR, 1.36; 95% CI, 1.13-1.63) among those with socioeconomic risk factors. Wine preference (>80% of alcohol from wine) and drinking with meals showed small protective associations with mortality, especially from cancer, but only in drinkers with socioeconomic or health-related risk factors and was associated with attenuating the excess mortality associated with high-, moderate- and even low-risk drinking.

**Conclusions and Relevance:**

In this cohort study of older drinkers from the UK, even low-risk drinking was associated with higher mortality among older adults with health-related or socioeconomic risk factors. The attenuation of mortality observed for wine preference and drinking only during meals requires further investigation, as it may mostly reflect the effect of healthier lifestyles, slower alcohol absorption, or nonalcoholic components of beverages.

## Key Points

**Question:**

Do health-related or socioeconomic risk factors modify the associations of alcohol consumption patterns with mortality among older drinkers?

**Findings:**

This cohort study in 135 103 older drinkers found that even low-risk drinking was associated with higher mortality among older adults with health-related or socioeconomic risk factors. Wine preference and drinking only with meals were associated with attenuating the excess mortality associated with alcohol consumption.

**Meaning:**

This cohort study identified inequalities in the detrimental health outcomes associated with alcohol that should be addressed to reduce the high disease burden of alcohol use.

## Teaser

This cohort study examines the associations of alcohol consumption patterns with 12-year mortality and the potential modification of these associations by health-related or socioeconomic risk factors.

## Introduction

Alcohol consumption is a leading cause of morbidity and mortality, accounting for approximately 5.1% of the global burden of disease and 5.3% of all deaths and being responsible for significant social and economic losses, thus representing a major public health problem.^1^ Additionally, the assumed benefits of drinking low amounts of alcohol, especially on cardiovascular disease (CVD) mortality,^2,3,4^ are being questioned due to selection biases, reverse causation, and residual confounding,^5^ supporting health messaging that the safest level of drinking is no drinking at all or less is better.^6,7^ Selection biases are often overlooked, but they can lead to a systematic underestimation of alcohol-related burden. That is the case of the abstainer bias, whereby the apparently lower mortality of light drinkers compared with abstainers could be explained by the higher death risk of the abstainers because they include former drinkers who quit alcohol due to poor health, as well as lifetime abstainers,^5^ who often have worse lifestyle and health characteristics than regular drinkers.^8^ Also, the healthy drinker/survivor bias, caused by overrepresentation of healthier drinkers who have survived the deleterious effects of alcohol, can distort comparisons, especially in older age.^5^ In addition, drinking habits may influence the association between the amount of alcohol consumed and health. In this context, wine preference has been associated with lower risk of death,^9^ CVD morbimortality,^10^ and diabetes,^11^ attributing the beneficial associations of wine to its high content in polyphenols.^12^ Furthermore, drinking with meals has been associated with lower risk of all-cause, non-CVD, and cancer deaths^13^ and frailty,^14^ so this might be a safer option for alcohol drinkers along with moderate consumption.^15^

The health impact of alcohol consumption may be greater in individuals with socioeconomic or health-related risk factors. On one hand, older adults with health-related risk factors are more susceptible to the harmful outcomes associated with alcohol due to their greater morbidity, higher use of alcohol-interacting drugs, and reduced tolerance.^16,17^ However, some studies have observed benefits of alcohol on unhealthy aging or frailty, especially of light alcohol intake^18,19^ and of a Mediterranean alcohol drinking pattern, defined as moderate alcohol consumption, preferably wine and accompanying meals,^14,20^ suggesting that the protective associations of these potentially beneficial drinking patterns might be greater in individuals with ill health, although they might be due to the aforementioned methodological issues.^5^ Therefore, it would be of interest to examine whether health-related risk factors modify the associations between alcohol consumption patterns and mortality.

On the other hand, there is evidence that socioeconomically disadvantaged populations have higher rates of alcohol-related harms for equivalent and even lower amounts of alcohol, probably due to the coexistence of other health challenges, including less healthy lifestyles, and lower social support or access to health care.^21,22^ Also, the potentially beneficial associations of wine preference and drinking during meals might be more important in individuals with socioeconomic risk factors. However, to our knowledge, no previous research has examined whether socioeconomic status modifies the associations between these potentially beneficial drinking patterns and health.

Therefore, the aim of our study is to examine the associations of several potentially beneficial alcohol consumption patterns, that is, consumption of low amounts of alcohol, wine preference, and drinking only during meals, with all-cause, cancer, and CVD mortality in older adults and their modification by health-related or socioeconomic risk factors, while addressing the main methodological issues deemed to bias such associations. Thus, we restrict analyses to current drinkers and use occasional drinkers instead of abstainers as the reference group to prevent selection biases, exclude deaths in the first 2 years of follow-up to reduce reverse causation, and adjust analyses for many sociodemographic, lifestyle, and clinical variables to palliate residual confounding. We also restrict analyses to older adults because most deaths occur in this population group, which also has a high prevalence of health-related risk factors and because the protective associations of alcohol consumption have been specifically observed in older adults,^6^ which is consistent with our aim to study potentially beneficial drinking patterns.

## Methods

This cohort study was approved by the North West Multi-Centre Research Ethics Committee, and all participants provided written informed consent before enrollment. This study is reported following the Strengthening the Reporting of Observational Studies in Epidemiology (STROBE) reporting guideline.

### Study Design and Participants

We used data from the UK Biobank cohort, a multicenter, prospective, population-based study with more than 500 000 participants aged 40 to 69 years identified from National Health Service primary care registers and enrolled at 22 assessment sites across England, Scotland, and Wales between 2006 and 2010. At the baseline assessment visit, they completed a computer-assisted interview and a touch-screen questionnaire on sociodemographic, lifestyle, and clinical characteristics, provided biological samples, and underwent physical and medical examinations. They were followed-up for mortality through linkage to national death registries. Additional information on the UK Biobank study has been reported elsewhere.^23,24^

### Study Variables

#### Alcohol Consumption Patterns

At the baseline assessment visit, study participants were asked about the frequency and mean amount of the main types of alcoholic beverages that they consumed, and alcohol content was estimated by multiplying the volume ingested (in milliliters) by the volume percentage of alcohol (4.5% for beer and cider, 11.5% for white and sparkling wine, 13% for red wine, 20% for fortified wine, and 40% for spirits) and by the specific gravity of ethanol (0.789 g/mL). According to their mean alcohol intake, drinking patterns were classified into occasional (≤2.86 g/d), low risk (men: >2.86-20.00 g/d; women: >2.86-10.00 g/d), moderate risk (men: >20.00-40.00 g/d; women: >10-20.00 g/d), and high risk (men: >40.00 g/d; women: >20.00 g/d), a categorization based on the recommendations from health authorities that we have used in previous studies.^25,26,27^ When more than 80% of alcohol came from a certain type of beverage, drinkers were classified as with preference for wine, with preference for other drinks, or with no preference.^27^ Participants were also classified as drinkers only during meals and as drinkers either only outside of meals or at any time. Finally, participants were classified as drinkers with no wine preference nor drinking only during meals, drinkers with wine preference or drinking only during meals, and drinkers with wine preference and drinking only during meals.

#### Health-Related Risk Factors

Health-related risk was assessed at baseline using the frailty index (FI) developed specifically for the UK Biobank^28^ based on the procedure used by Rockwood et al.^29^ A total of 49 health deficits were considered, most dichotomously (1 point if present and 0 points otherwise), and a few according to severity (0 points for no deficit, 0.25-0.75 points for mild to moderate deficits, and 1 point for severe deficit). The FI score was calculated as the total sum of points assigned to each health deficit divided by the number of deficits considered and ranged from 0.00 to 0.57. The complete list of health deficits and associated scores can be found in eTable 1 in Supplement 1. Participants were considered to have health-related risk factors if they were prefrail or frail (FI > 0.12).^28^

#### Socioeconomic Risk Factors

Socioeconomic risk was assessed at baseline using the Townsend deprivation index (TDI),^30^ which measures the level of an area’s socioeconomic deprivation. TDI ranges from −6.26 to 10.16, with higher score indicating greater deprivation. Participants were considered to have socioeconomic risk factors if they lived in more deprived areas (TDI > 0) and not if they lived in more affluent areas (TDI ≤ 0).

#### All-Cause and Cause-Specific Mortality

Information on mortality was obtained from death certificates held by the National Health Service (NHS) Information Centre (NHS England) up to September 30, 2021, for participants in England and Wales, and by the NHS Central Register Scotland (National Records of Scotland) up to October 31, 2021, for participants in Scotland.^31,32^ Length of follow-up was estimated as the time from the baseline assessment visit to the date of death or administrative censoring, whichever came first. Cause-specific mortality was ascertained with the *International Statistical Classification of Diseases and Related Health Problems, Tenth Revision* (*ICD-10*) classification^33^: codes C00 to C97 as primary cause of death for cancer and codes I00 to I99 for CVD.

#### Potential Confounders

We also used baseline information on sociodemographic, lifestyle, and clinical characteristics, including sex, age, self-reported race and ethnicity, education (college or university degree; A levels, AS levels, or equivalent; O levels, General Certificate of Secondary Education, or equivalent; Certificate of Secondary Education or equivalent; National Vocational Qualification, Higher National Diploma, Higher National Certificate, or equivalent; other professional qualifications; and no qualifications), tobacco smoking (never, former, or current), leisure-time physical activity (metabolic equivalents of task-hours per week), time spent watching television (hours per day), and prevalent morbidities (diabetes, CVD, and cancer) that could have a potential effect on the amount of alcohol consumed. In the UK Biobank, race and ethnicity are classified as Asian (Indian, Pakistani, Bangladeshi, any other Asian background), Black (Caribbean, African, any other Black background), Chinese, multiple (White and Black Caribbean, White and Black African, White and Asian, any other mixed background), White (British, Irish, any other White background), and other (any group not specified, eg, Arab).

### Statistical Analysis

From 217 462 participants aged at least 60 years in the UK Biobank cohort, we excluded 36 284 with incomplete information on alcohol consumption, 10 456 never drinkers, 8295 former drinkers, and 20 167 known binge drinkers (those who consumed ≥6 units of alcohol in 1 session) to avoid classifying binge drinkers with low mean alcohol intake as low-risk drinkers. We additionally excluded 1140 participants who died in the first 2 years of follow-up and 6017 participants with missing information on the FI (194 participants), the TDI (116 participants), and potential confounders (5707 participants). Thus, the analytical sample included 135 103 individuals.

The associations of alcohol consumption patterns (mean alcohol intake status, wine preference, and drinking during meals) at baseline with all-cause and cause-specific mortality were summarized with hazard ratios (HRs) and their 95% CIs obtained from Cox regression; the models included interactions between alcohol consumption patterns and health-related or socioeconomic risk factors and adjusted for baseline sociodemographic (sex, age, race and ethnicity, education, and TDI [except when stratifying by socioeconomic risk factors]), lifestyle (tobacco smoking, leisure-time physical activity, and time spent watching television), and clinical characteristics (diabetes, CVD, cancer, and FI score [except when stratifying by health-related risk factors]) of study participants. Analyses of alcohol intake were further adjusted for wine preference and drinking during meals, whereas analyses of wine preference and drinking during meals were further adjusted for mean alcohol intake and the other drinking pattern.

To characterize whether wine preference and drinking during meals modified the association of mean alcohol intake with mortality, we tested interaction terms defined as the product of the categories of mean alcohol intake by 3 categories of drinking patterns (no wine preference nor drinking only during meals, wine preference or drinking only during meals, and wine preference and drinking only during meals).

Additionally, we assessed whether sociodemographic and lifestyle variables modified the study associations by testing interaction terms defined as the product of alcohol consumption patterns by categories of such variables (except mean alcohol intake status by sex, as sex was included in the definition of alcohol intake status). Since no interactions were found, the results are presented for the total sample. Finally, we performed additional sensitivity analyses excluding participants with prevalent cancer at baseline for cancer mortality or those with prevalent CVD at baseline for CVD mortality.

Statistical significance was set at 2-sided *P* < .05. Analyses were performed with Stata software version 17 (StataCorp). Data were analyzed from September 2023 to May 2024.

## Results

A total of 135 103 participants (median [IQR] age, 64.0 [62.0-67.0] years; 67 693 [50.1%] women) were included. Occasional drinkers less often identified as White; were more frequently residents in England, women, and never smokers; were less physically active; had a lower educational level, a lower prevalence of CVD; and had a higher prevalence of diabetes, cancer, and health-related risk factors. Having socioeconomic risk factors was less frequent in low- and moderate-risk drinkers (Table 1).

**Table 1.** Baseline Characteristics of Older Drinkers From the UK Biobank Cohort by Categories of Mean Alcohol Intake Status

Characteristic	Participants by mean drinking habits, No. (%)^a^			
	Occasional (n = 12 049)	Low risk (n = 56 015)	Moderate risk (n = 41 674)	High risk (n = 25 365)
Region of the assessment center				
England	11 625 (96.5)	50 326 (89.8)	37 124 (89.1)	22 550 (88.9)^b^
Scotland	153 (1.3)	3525 (6.3)	2912 (6.7)	1817 (6.2)
Wales	271 (2.2)	2164 (3.9)	1638 (3.9)	998 (3.9)
Sex				
Women	8766 (72.3)	25 510 (45.5)	21 260 (51.0)	12 157 (47.9)
Men	3283 (27.2)	30 505 (54.5)	20 414 (49.0)	13 208 (52.1)^b^
Age, median (IQR), y	64.0 (62.0 to 67.0)	64.0 (62.0 to 67.0)	64.0 (62.0 to 66.0)	64.0 (62.0 to 66.0)^b^
Race and ethnicity^c^				
Asian	250 (2.1)	520 (0.9)	199 (0.5)	84 (0.3)
Black	228 (1.9)	367 (0.7)	124 (0.3)	43 (0.2)
Chinese	39 (0.3)	65 (0.1)	15 (0.1)	7 (0.1)
Multiple	61 (0.5)	520 (0.3)	102 (0.2)	46 (0.1)
White	11 333 (94.1)	54 700 (97.7)	41 130 (98.7)	25 146 (99.1)^b^
Other	138 (1.1)	216 (0.4)	104 (0.2)	39 (0.2)
With college or university degree	2765 (22.9)	15 698 (28.0)	11 485 (27.6)	6320 (24.9)^b^
Never smoker	7559 (62.7)	31 489 (56.2)	18 636 (44.7)	7884 (31.1)^b^
Physical activity, median (IQR), MET-h/week	596 (188 to 1313)	795 (295 to 1718)	844 (323 to 1808)	771 (253 to 1796)^b^
Time watching television, median (IQR), h/d	3.0 (2.0 to 4.0)	3.0 (2.0 to 4.0)	3.0 (2.0 to 4.0)	3.0 (2.0 to 4.0)^b^
Diabetes	965 (8.0)	3619 (6.5)	2209 (5.3)	1449 (5.7)^b^
Cardiovascular disease	975 (8.1)	5059 (9.0)	3667 (8.8)	2270 (8.9)^b^
Cancer	1425 (11.8)	5796 (10.3)	4443 (10.7)	2721 (10.7)^b^
Alcohol intake, median (IQR), g/d	1.4 (0.8 to 1.9)	8.1 (5.5 to 12.0)	19.5 (13.5 to 27.8)	43.2 (27.1 to 57.6)^b^
Frailty index, median (IQR)^d^	0.13 (0.08 to 0.18)	0.11 (0.07 to 0.17)	0.11 (0.07 to 0.16)	0.12 (0.08 to 0.17)^b^
Townsend deprivation index, median (IQR)^e^	−2.1 (−3.5 to 0.3)	−2.6 (−3.9 to −0.6)	−2.6 (−3.9 to −0.5)	−2.2 (−3.7 to 0.3)^b^
With health-related risk factors, median (IQR)^f^	6543 (54.3)	25 985 (46.4)	19 344 (46.4)	12 806 (50.5)^b^
With socioeconomic risk factors^g^	3284 (27.3)	11 768 (21.0)	9076 (21.8)	6946 (27.4)^b^

Abbreviation: MET, metabolic equivalent of task.

^a^ Occasional drinkers consumed 20 g/week or less; low-risk drinkers, more than 20 g/week to 20 g/d for men and more than 20 g/week to 10 g/d for women; moderate-risk drinkers, more than 20 to 40 g/d for men and more than 10 to 20 g/d for women; high-risk drinkers, more than 40 g/d for men and more than 20 g/d for women.

^b^ *P* < .05.

^c^ In the UK Biobank, race and ethnicity are classified as Asian (Indian, Pakistani, Bangladeshi, any other Asian background), Black (Caribbean, African, any other Black background), Chinese, multiple (White and Black Caribbean, White and Black African, White and Asian, any other mixed background), White (British, Irish, any other White background), and other (any group not specified, eg, Arab).

^d^ A higher score indicates greater health-related risk factors.

^e^ A higher score indicates greater socioeconomic risk factors (greater levels of an area’s socioeconomic deprivation).

^f^ Frailty index score greater than 0.12.

^g^ Townsend deprivation index score greater than 0.

Over a median (range) follow-up of 12.4 (2.0 to 14.8) years, 15 833 deaths were recorded, including 7871 cancer deaths and 3215 CVD deaths. Compared with occasional drinking, low-risk drinking was associated with higher cancer mortality (HR, 1.11; 95% CI, 1.01-1.22); moderate-risk drinking was associated with higher all-cause (HR, 1.10; 95% CI, 1.03-1.18) and cancer (HR, 1.15; 95% CI, 1.05-1.27) mortality; and high-risk drinking was associated with higher all-cause (HR, 1.33; 95% CI, 1.24-1.42), cancer (HR, 1.39; 95% CI, 1.26-1.53), and CVD (HR, 1.21; 95% CI, 1.04-1.41) mortality (Table 2). Hazards were greater in individuals with health-related or socioeconomic risk factors vs those without across categories of alcohol intake. Interestingly, while no associations with mortality were found in participants without health-related or socioeconomic risk factors among low- or moderate-risk drinkers, low-risk drinkers with health-related risk factors had higher cancer mortality (HR, 1.15; 95% CI, 1.01-1.30) and moderate-risk drinkers with health-related risk factors had higher all-cause (HR, 1.10; 95% CI, 1.01-1.19) and cancer (HR, 1.19; 95% CI, 1.05-1.35) mortality (Table 2). Likewise, both low-risk and moderate-risk drinkers with socioeconomic risk factors showed higher mortality from all causes (low risk: HR, 1.14; 1.01-1.28; moderate risk: 1.17; 95% CI, 1.03-1.32) and cancer (low-risk: HR, 1.25; 95% CI, 1.04-1.50; moderate risk: HR, 1.36; 95% CI, 1.13-1.63) (Table 2).

**Table 2.** Association of Mean Alcohol Intake Status With Mortality in Older Drinkers From the UK Biobank Cohort

Alcohol intake status^a^	All-cause mortality			Cancer mortality			CVD mortality		
	Deaths, No./total No.	HR (95% CI)^b^	*P* value for interaction	Deaths, No./total No.	HR (95% CI)^b^	*P* value for interaction	Deaths, No./total No.	HR (95% CI)^b^	*P* value for interaction
Occasional	1097/12 049	1 [Reference]	NA	526/12 045	1 [Reference]	NA	232/12 045	1 [Reference]	NA
Low risk	6114/56 015	1.06 (1.00-1.13)	NA	3012/55 988	1.11 (1.01-1.22)^c^	NA	1273/55 988	0.97 (0.84-1.11)	NA
Moderate risk	4789/41 674	1.10 (1.03-1.18)^d^	NA	2418/41 652	1.15 (1.05-1.27)^d^	NA	926/41 652	0.95 (0.82-1.10)	NA
High risk	3833/25 365	1.33 (1.24-1.42)^e^	NA	1915/25 353	1.39 (1.26-1.53)^e^	NA	784/25 353	1.21 (1.04-1.41)^c^	NA
**Health-related risk factors**									
No									
Occasional	397/5506	1 [Reference]	NA	220/5504	1 [Reference]	NA	74/5504	1 [Reference]	NA
Low risk	2607/30 030	1.02 (0.92-1.14)	NA	1434/30 014	1.05 (0.91-1.21)	NA	470/30 014	0.90 (0.70-1.15)	NA
Moderate risk	2054/22 330	1.08 (0.97-1.21)	NA	1143/22 324	1.10 (0.95-1.27)	NA	362/22 324	0.96 (0.74-1.23)	NA
High risk	1409/12 559	1.21 (1.08-1.36)^e^	NA	797/12 554	1.26 (1.08-1.46)^d^	NA	264/12 554	1.14 (0.88-1.48)	NA
Yes^f^									
Occasional	700/6543	1 [Reference]	.04	306/6541	1 [Reference]	.30	158/6541	1 [Reference]	.51
Low risk	3507/25 985	1.08 (0.99-1.17)	.44	1578/25 974	1.15 (1.01-1.30)^c^	.36	803/25 974	0.99 (0.84-1.18)	.51
Moderate risk	2735/19 344	1.10 (1.01-1.19)^c^	.83	1275/19 328	1.19 (1.05-1.35)^d^	.43	564/19 328	0.93 (0.78-1.12)	.87
High risk	2424/12 806	1.39 (1.28-1.52)^e^	.05	1118/12 799	1.49 (1.31-1.69)^e^	.10	520/12 799	1.24 (1.03-1.48)^c^	.60
**Socioeconomic risk factors**									
No									
Occasional	756/8765	1 [Reference]	NA	383/8761	1 [Reference]	NA	153/8761	1 [Reference]	NA
Low risk	4517/44 247	1.02 (0.95-1.11)	NA	2291/44 225	1.05 (0.94-1.17)	NA	909/44 225	0.92 (0.78-1.10)	NA
Moderate risk	3461/32 598	1.07 (0.98-1.15)	NA	1771/32 581	1.08 (0.97-1.21)	NA	666/32 581	0.95 (0.79-1.13)	NA
High risk	2457/18 419	1.26 (1.16-1.37)^e^	NA	1294/18 410	1.31 (1.17-1.47)^e^	NA	468/18 410	1.12 (0.93-1.35)	NA
Yes^g^									
Occasional	341/3284	1 [Reference]	.18	143/3284	1 [Reference]	.21	79/3284	1 [Reference]	.17
Low risk	1597/11 768	1.14 (1.01-1.28)^c^	.13	721/11 763	1.25 (1.04-1.50)^c^	.11	364/11 763	1.04 (0.81-1.33)	.44
Moderate risk	1328/9076	1.17 (1.03-1.32)^c^	.21	647/9071	1.36 (1.13-1.63)^d^	.04	260/9071	0.93 (0.72-1.20)	.92
High risk	1376/6946	1.47 (1.31-1.66)^e^	.04	621/6943	1.59 (1.32-1.91)^e^	.08	316/6943	1.37 (1.07-1.76)^c^	.20

Abbreviations: HR, hazard ratio; NA, not applicable.

^a^ Occasional drinkers consumed 20 g/week or less; low-risk drinkers, more than 20 g/week to 20 g/d for men and more than 20 g/week to 10 g/d for women; moderate-risk drinkers, more than 20 to 40 g/d for men and more than 10 to 20 g/d for women; high-risk drinkers, more than 40 g/d for men and more than 20 g/d for women.

^b^ Cox regression model adjusted for sex, age, race and ethnicity, education, region of the assessment center, smoking status (never, former, or current), physical activity (metabolic equivalent task hours per week, in tertiles), television watching time (hours per day, in tertiles), diabetes, cardiovascular disease, cancer, Townsend deprivation index score (except when stratifying by socioeconomic risk factors), frailty index score (except when stratifying by health-related risk factors), wine preference, and drinking during meals.

^c^ *P* < .05.

^d^ *P* < .01.

^e^ *P* < .001.

^f^ Frailty index score greater than 0.12.

^g^ Townsend deprivation index score greater than 0.

Wine preference and drinking only during meals were associated with lower all-cause mortality only in participants with health-related risk factors (wine preference: HR, 0.92; 95% CI, 0.87-0.97; drinking only during meals: HR, 0.93; 95% CI, 0.89-0.97), as well as in participants with socioeconomic risk factors (wine preference: HR, 0.84; 95% CI, 0.78-0.90; drinking only during meals: HR, 0.83; 95% CI, 0.78-0.89) (Table 3). Drinking only during meals was also associated with lower cancer mortality in participants with health-related risk factors (HR, 0.92; 95% CI, 0.86-0.99) or socioeconomic risk factors (HR, 0.85; 95% CI, 0.78-0.94) (Table 3). Furthermore, in individuals with socioeconomic risk factors, wine preference was associated with lower cancer mortality (HR, 0.89; 95% CI, 0.80-0.99) and drinking only during meals with lower CVD mortality (HR, 0.86; 95% CI, 0.75-1.00) (Table 3). Adhering to both drinking patterns was associated with lower all-cause, cancer, and CVD mortality in drinkers with health-related or socioeconomic risk factors, and to a lesser extent, with lower all-cause death in drinkers without health-related risk factors (eTable 2 in Supplement 1). Importantly, wine preference and drinking during meals modified the association of mean alcohol intake with mortality: the excess risk of all-cause, cancer, and CVD death for high-risk drinkers, of all-cause and cancer death for moderate-risk drinkers, and of cancer death for low-risk drinkers vs occasional drinkers was attenuated and even lost among individuals with these drinking patterns (Table 4). Analyses excluding participants with prevalent cancer at baseline for cancer mortality, or those with prevalent CVD at baseline for CVD mortality showed consistent results (eTables 3-6 in Supplement 1).

**Table 3.** Association of Wine Preference and Drinking During Meals With Mortality in Older Drinkers From the UK Biobank Cohort

Drinking behavior	All-cause mortality			Cancer mortality			CVD mortality		
	Deaths, No./total No.	HR (95% CI)^a^	*P* value for interaction	Deaths, No./total No.	HR (95% CI)^a^	*P* value for interaction	Deaths, No./total No.	HR (95% CI)^a^	*P* value for interaction
Wine preference									
No wine preference	11410/84 625	1 [Reference]	NA	5437/84 575	1 [Reference]	NA	2432/84 575	1 [Reference]	NA
Wine preference	4423/50 478	0.93 (0.90-0.97)^b^		2434/50 463	0.98 (0.93-1.04)		783/50 463	0.92 (0.84-1.01)	
**By health-related risk factors**									
No			.37						.26
No wine preference	4422/42 230	1 [Reference]		2367/42 212	1 [Reference]	.44	850/42 212	1 [Reference]	
Wine preference	2045/28 195	0.95 (0.90-1.00)		1227/28 184	1.00 (0.93-1.08)		320/28 184	0.87 (0.76-1.00)	
Yes^c^									
No wine preference	6988/42 395	1 [Reference]		3070/42 363	1 [Reference]		1582/42 363	1 [Reference]	
Wine preference	2378/22 283	0.92 (0.87-0.97)^b^		1207/22 279	0.96 (0.90-1.04)		463/22 279	0.95 (0.85-1.06)	
**By socioeconomic risk factors**									
No			.001			.03			.64
No wine preference	7753/63 640	1 [Reference]		3819/63 600	1 [Reference]		1607/63 600	1 [Reference]	
Wine preference	3438/40 389	0.96 (0.92-1.01)		1920/40 377	1.01 (0.95-1.07)		589/40 377	0.93 (0.84-1.03)	
Yes^d^									
No wine preference	3657/20 985	1 [Reference]		1618/20 975	1 [Reference]		825/20 975	1 [Reference]	
Wine preference	985/10 089	0.84 (0.78-0.90)^b^		514/10 086	0.89 (0.80-0.99)^e^		194/10 086	0.89 (0.75-1.05)	
Drinking during meals									
No drinking only during meals	9160/66 297	1 [Reference]		4423/66 260	1 [Reference]		1918/66 260	1 [Reference]	
Drinking only during meals	6673/68 806	0.94 (0.91-0.97)^b^		3448/68 778	0.93 (0.88-0.98)^f^		1297/68 778	0.96 (0.89-1.04)	
**By health-related risk factors**									
No			.40			.76			.57
No drinking only during meals	3394/32 074	1 [Reference]		1859/32 058	1 [Reference]		622/32 058	1 [Reference]	
Drinking only during meals	3073/38 351	0.95 (0.90-1.00)		1735/38 338	0.94 (0.87-1.00)		548/38 338	0.99 (0.87-1.11)	
Yes^c^									
No drinking only during meals	5766/34 223	1 [Reference]		2564/34 202	1 [Reference]		1296/34 202	1 [Reference]	
Drinking only during meals	3600/30 455	0.93 (0.89-0.97)^b^		1713/30 440	0.92 (0.86-0.99)^e^		749/30 440	0.95 (0.86-1.04)	
**By socioeconomic risk factors**									
No			<.001			.04			.08
No drinking only during meals	5922/48 241	1 [Reference]		2984/48 211	1 [Reference]		1193/48 211	1 [Reference]	
Drinking only during meals	5269/55 788	0.97 (0.94-1.01)		2755/55 766	0.95 (0.90-1.01)		1003/55 766	1.00 (0.91-1.09)	
Yes^d^									
No drinking only during meals	3238/18 056	1 [Reference]		1439/18 049	1 [Reference]		725/18 049	1 [Reference]	
Drinking only during meals	1404/13 018	0.83 (0.78-0.89)^b^		693/13 012	0.85 (0.78-0.94)^f^		294/13 012	0.86 (0.75-1.00)^e^	

Abbreviations: CVD, cardiovascular disease; HR, hazard ratio; NA, not applicable.

^a^ Cox regression model adjusted for sex, age, race and ethnicity, education, region of the assessment center, smoking status (never, former, or current), physical activity (metabolic equivalent task hours per week, tertiles), television watching time (hours per day, tertiles), diabetes, cardiovascular disease, cancer, Townsend deprivation index, frailty index score, mean alcohol intake (grams per day, quintiles), and the other drinking pattern.

^b^ *P* < .001.

^c^ Frailty index score greater than 0.12.

^d^ Townsend deprivation index score greater than 0.

^e^ *P* < .05.

^f^ *P* < .01.

**Table 4.** Association of Mean Alcohol Intake Status With Mortality in Older Drinkers From the UK Biobank Cohort, by Drinking Patterns

Alcohol intake status^a^	All-cause mortality			Cancer mortality			CVD mortality		
	Deaths, No./total No.	HR (95% CI)^b^^,^^c^	P value for interaction	Deaths, No./total No.	HR (95% CI)^b^^,^^d^	P value for interaction	Deaths, No./total No.	HR (95% CI)^b^^,^^e^	P value for interaction
**No wine preference nor drinking during meals**									
Occasional	354/3354	1 [Reference]	NA	154/3353	1 [Reference]	NA	80/3353	1 [Reference]	NA
Low risk	2567/19 770	1.10 (0.98-1.23)	.89	1201/19 758	1.20 (1.01-1.42)^f^	.60	567/19 758	0.99 (0.78-1.26)	.43
Moderate risk	2461/16 948	1.17 (1.05-1.31)^g^	.36	1184/16 934	1.31 (1.10-1.55)^g^	.14	501/16 934	0.98 (0.77-1.24)	.80
High risk	2482/12 721	1.49 (1.33-1.67)^h^	.001	1167/12 715	1.63 (1.38-1.94)^h^	.02	551/12 715	1.35 (1.07-1.72)^f^	.007
**Wine preference or drinking during meals**									
Occasional	377/4054	1 [Reference]	NA	178/4052	1 [Reference]	NA	73/4052	1 [Reference]	NA
Low risk	2136/20 185	1.06 (0.95-1.18)	NA	1056/20 176	1.10 (0.94-1.30)	NA	448/20 176	1.08 (0.84-1.38)	NA
Moderate risk	1433/13 792	1.09 (0.97-1.22)	NA	741/13 786	1.15 (0.97-1.35)	NA	262/13 786	1.02 (0.79-1.33)	NA
High risk	896/7305	1.26 (1.11-1.42)^h^	NA	473/7301	1.32 (1.11-1.57)^g^	NA	169/7301	1.27 (0.97-1.68)	NA
**Wine preference and drinking during meals**									
Occasional	366/4641	1 [Reference]	NA	194/4640	1 [Reference]	NA	79/4640	1 [Reference]	NA
Low risk	1411/16 060	1.07 (0.95-1.20)	NA	755/16 054	1.07 (0.91-1.25)	NA	258/16 054	0.85 (0.66-1.10)	NA
Moderate risk	895/10934	1.04 (0.92-1.18)	NA	493/10 932	1.03 (0.87-1.22)	NA	163/10 932	0.90 (0.69-1.18)	NA
High risk	455/5339	1.06 (0.92-1.22)	NA	275/5337	1.13 (0.94-1.37)	NA	64/5337	0.72 (0.52-1.01)	NA

Abbreviations: CVD, cardiovascular disease; HR, hazard ratio; NA, not applicable.

^a^ Occasional drinkers consumed 20 g/week or less; low-risk drinkers, more than 20 g/week to 20 g/d for men and more than 20 g/week to 10 g/d for women; moderate-risk drinkers, more than 20 to 40 g/d for men and more than 10 to 20 g/d for women; high-risk drinkers, more than 40 g/d for men and more than 20 g/d for women.

^b^ Cox regression model adjusted for sex, age, ethnicity, education, region of the assessment center, smoking status (never, former, or current), physical activity (METs-h/week, tertiles), TV watching time (h/d, tertiles), diabetes, cardiovascular disease, cancer, Townsend deprivation index score, and frailty index score.

^c^ Overall *P* for interaction < .001.

^d^ Overall *P* for interaction = .03.

^e^ Overall *P* for interaction = .02.

^f^ *P* < .05.

^g^ *P* < .01.

^h^ *P* < .001.

## Discussion

This cohort study in older alcohol drinkers from the UK found that compared with occasional drinkers, low-risk drinkers had higher cancer mortality, moderate-risk drinkers had higher all-cause and cancer mortality, and high-risk drinkers had higher all-cause, cancer, and CVD mortality. The excess mortality associated with alcohol consumption was higher in individuals with health-related and socioeconomic risk factors, among whom even low-risk drinkers had higher mortality, especially from cancer. Wine preference and drinking only with meals showed small protective associations with mortality, especially from cancer, among drinkers with health-related and socioeconomic risk factors, and these 2 drinking patterns attenuated the excess mortality associated with high-, moderate-, and even low-risk drinking.

In line with recent research on the associations between alcohol use and health,^6,34,35^ our results corroborate the detrimental outcomes associated with heavy drinking in older adults. However, we also found higher risk for all-cause and cancer deaths in moderate-risk drinkers, unlike most previous research, which has reported protective associations of low to moderate alcohol consumption, mainly for all-cause^2,3,4,36^ and CVD^3,36,37^ mortality, ischemic heart disease,^3,6,34^ and diabetes,^6^ or null associations with all-cause mortality,^38^ CVD,^39^ and unhealthy aging.^20^ This discrepancy may be due to the implementation of an important methodological improvement in our analyses, that is, using occasional drinkers as the reference group instead of lifetime abstainers, to prevent selection bias caused by misclassification of former drinkers as abstainers, and to palliate residual confounding because they are more like light drinkers than are never drinkers.^40,41^ In fact, another analysis of the UK Biobank cohort that also avoided selection biases found an increased CVD risk in the general population for drinking up to 14 units per week.^42^

To our knowledge, there are no studies examining the potential modification of health-related risk factors on the association between alcohol use and health. The stronger associations between mean alcohol intake and mortality observed in older adults with health-related risk factors make sense, since they have more morbid conditions potentially aggravated by alcohol and greater use of alcohol-interacting medications than their counterparts without health-related risk factors.^16,17^ The fact that even low-risk drinkers with these risk factors had higher risk of cancer death is an important finding, which is consistent with the reported increased risk of several types of cancer and cancer mortality even with very low amounts of alcohol.^6,36,37,43^

Our results also suggest that socioeconomic status acts as a modifier of the association between the amount of alcohol consumed and mortality, as mortality hazard was much greater in individuals with socioeconomic risk factors than in individuals without, in line with previous research.^21,22,44,45^ We even found a detrimental association of low amounts of alcohol with all-cause and cancer mortality in this group, unlike the MORGAM study by DiCasetnuovo et al^44^ reporting a lower mortality associated with consuming no more than 10 g/d of alcohol, which was clearer in individuals with higher vs lower education.^44^ These discrepant results could again be explained by the different reference groups used: occasional drinkers in our study and never drinkers in the MORGAM study. Importantly, although older adults with socioeconomic risk factors have a higher risk of ill health and death, probably due to the coexistence of other health challenges, especially poorer lifestyles,^21,22^ the observed associations in our study were independent of lifestyles, suggesting that other factors should account for them.

Regarding the potentially beneficial drinking patterns, that is, wine preference and drinking during meals, the literature is inconsistent. A 2018 pool of studies^34^ reported a nondifferential association of specific types of alcoholic drinks with all-cause mortality and several CVD outcomes, whereas other studies have found protective health associations for wine but not other beverages.^15,46^ Drinking with meals has also shown protective associations with several health outcomes.^15^ In our analysis, these drinking patterns modified the association between alcohol intake and death risk. On one hand, the protective association for mortality of these patterns was only observed in individuals with socioeconomic or health-related risk factors, independently of the amount of alcohol consumed. On the other hand, the detrimental association of alcohol intake was more evident in individuals without these patterns. These findings suggest that the less detrimental associations of alcohol intake from wine or during meals are not due to alcohol itself, but to other factors, including nonalcoholic components of wine, such as antioxidants, slower absorption of alcohol ingested with meals and its consequent reduced alcoholaemia, as well as spacing drinks when drinking only with meals, or more moderate attitudes in individuals who choose to adhere to these drinking patterns.

### Strengths and Limitations

Our study has several strengths, such as the large sample size, the long follow-up, and the methodological improvements implemented to prevent selection biases and reduce reverse causation. However, it also has some limitations. First, alcohol intake was self-reported, and therefore prone to some degree of misclassification. Also, alcohol intake was measured only at baseline and not at multiple time points over the life span, not allowing us to take into account changes in alcohol intake before the baseline assessment or to redistribute former drinkers among categories of current drinkers to reduce selection bias; this may have led to an underestimation of the true effects of alcohol consumption.^5^ Second, as in any observational study, we cannot entirely rule out residual confounding, despite adjusting for many potential confounders. And third, this study was conducted in older adults in the UK with a high proportion of White participants, so our results may not be generalizable to other racial ethnic groups or populations with different lifestyles, drinking patterns, or socioeconomic development.

## Conclusions

This cohort study among older drinkers from the UK did not find evidence of a beneficial association between low-risk alcohol consumption and mortality; however, we observed a detrimental association of even low-risk drinking in individuals with socioeconomic or health-related risk factors, especially for cancer deaths. The attenuation of the excess mortality associated with alcohol among individuals who preferred to drink wine or drink only during meals requires further investigation to elucidate the factors that may explain it. Finally, these results have important public health implications because they identify inequalities in the detrimental health outcomes associated with alcohol that should be addressed to reduce the high burden of disease of alcohol use.

## Notes

Supplement 1. **eTable 1.** Health Deficits of the Frailty Index in the UK Biobank Cohort **eTable 2.** Association of Wine Preference and Drinking During Meals With Mortality in Older Drinkers From the UK Biobank Cohort **eTable 3.** Association of Average Alcohol Intake Status With Mortality in Older Drinkers From the UK Biobank Cohort, Excluding Participants With Prevalent Cancer at Baseline for Cancer Mortality, or Those With Prevalent CVD at Baseline for CVD Mortality **eTable 4.** Association of Wine Preference or Drinking During Meals With Mortality in Older Drinkers From the UK Biobank Cohort, Excluding Participants With Prevalent Cancer at Baseline for Cancer Mortality, or Those With Prevalent CVD at Baseline for CVD Mortality **eTable 5.** Association of Wine Preference and Drinking During Meals With Mortality in Older Drinkers From the UK Biobank Cohort, Excluding Participants With Prevalent Cancer at Baseline for Cancer Mortality, or Those With Prevalent CVD at Baseline for CVD Mortality **eTable 6.** Association of Average Alcohol Intake Status With Mortality in Older Drinkers From the UK Biobank Cohort, by Drinking Patterns, Excluding Participants With Prevalent Cancer at Baseline for Cancer Mortality, or Those With Prevalent CVD at Baseline for CVD Mortality

Supplement 2.
Data Sharing Statement
