# Supplementary material for: Alcohol Consumption Patterns and Mortality Among Older Adults With Health-Related or Socioeconomic Risk Factors
Source: JAMA Netw Open. 2024 Aug 12;7(8):e2424495. doi: 10.1001/jamanetworkopen.2024.24495 (PMC11320169; doi:10.1001/jamanetworkopen.2024.24495)
Supplement: Supplement 1. — eTable 1. Health Deficits of the Frailty Index in the UK Biobank Cohort eTable 2. Association of Wine Preference and Drinking During Meals With Mortality in Older Drinkers From the UK Biobank Cohort eTable 3. Association of Average Alcohol Intake Status With Mortality in Older Drinkers From the UK Biobank Cohort, Excluding Participants With Prevalent Cancer at Baseline for Cancer Mortality, or Those With Prevalent CVD at Baseline for CVD Mortality eTable 4. Association of Wine Preference or Drinking During Meals With Mortality in Older Drinkers From the UK Biobank Cohort, Excluding Participants With Prevalent Cancer at Baseline for Cancer Mortality, or Those With Prevalent CVD at Baseline for CVD Mortality eTable 5. Association of Wine Preference and Drinking During Meals With Mortality in Older Drinkers From the UK Biobank Cohort, Excluding Participants With Prevalent Cancer at Baseline for Cancer Mortality, or Those With Prevalent CVD at Baseline for CVD Mortality eTable 6. Association of Average Alcohol Intake Status With Mortality in Older Drinkers From the UK Biobank Cohort, by Drinking Patterns, Excluding Participants With Prevalent Cancer at Baseline for Cancer Mortality, or Those With Prevalent CVD at Baseline for CVD Mortality [file jamanetwopen-e2424495-s001.pdf]

Supplemental Online Content

Ortolá R, Sotos-Prieto M, García-Esquinas E, Galán I, Rodríguez-Artalejo F. Alcohol consumption patterns and mortality among older adults with health-related or socioeconomic risk factors. *JAMA Netw Open*. 2024;7(8):e2424495. doi:10.1001/jamanetworkopen.2024.24495

eTable 1. Health Deficits of the Frailty Index in the UK Biobank Cohort

eTable 2. Association of Wine Preference and Drinking During Meals With Mortality in Older Drinkers From the UK Biobank Cohort

eTable 3. Association of Mean Alcohol Intake Status With Mortality in Older Drinkers From the UK Biobank Cohort, Excluding Participants With Prevalent Cancer at Baseline for Cancer Mortality, or Those With Prevalent CVD at Baseline for CVD Mortality

eTable 4. Association of Wine Preference or Drinking During Meals With Mortality in Older Drinkers From the UK Biobank Cohort, Excluding Participants With Prevalent Cancer at Baseline for Cancer Mortality, or Those With Prevalent CVD at Baseline for CVD Mortality

eTable 5. Association of Wine Preference and Drinking During Meals With Mortality in Older Drinkers From the UK Biobank Cohort, Excluding Participants With Prevalent Cancer at Baseline for Cancer Mortality, or Those With Prevalent CVD at Baseline for CVD Mortality

eTable 6. Association of Average Alcohol Intake Status With Mortality in Older Drinkers From the UK Biobank Cohort, by Drinking Patterns, Excluding Participants With Prevalent Cancer at Baseline for Cancer Mortality, or Those With Prevalent CVD at Baseline for CVD Mortality

This supplemental material has been provided by the authors to give readers additional information about their work.

© 2024 Ortolá R et al. *JAMA Network Open.*

eTable 1. Health deficits of the Frailty Index in the UK Biobank cohort**.**

	Health deficits	Scoring
1	Glaucoma *	No=0, Yes=1
2	Cataracts *	No=0, Yes=1
3	Hearing difficulty	No=0, Yes=1
4	Migraine *	No=0, Yes=1
5	Dental problems	No=0, Yes=1
6	Self-rated health	Excellent=0, Good=0.25, Fair=0.5, Poor=1
7	Fatigue: frequency of tiredness/lethargy in last 2 weeks	Not at all=0, Several days=0.25, More than half=0.5, Nearly every day=1
8	Sleep: experience of sleeplessness/insomnia	Never/rarely=0, Sometimes=0.5, Usually=1
9	Depressed feelings: frequency in last two weeks	Not at all=0, Several days=0.5, More than half=0.75, Nearly every day=1
10	Self-described nervous personality	No=0, Yes=1
11	Severe anxiety/panic attacks *	No=0, Yes=1
12	Common to feel loneliness	No=0, Yes=1
13	Sense of misery (ever/never)	No=0, Yes=1
14	Infirmity: long-standing illness or disability	No=0, Yes=1
15	Falls in last year	No falls=0, One fall=0.5, More than one=1
16	Fractures/broken bones in last five years	No=0, Yes=1
17	Diabetes *	No=0, Yes=1
18	Myocardial infarction *	No=0, Yes=1
19	Angina *	No=0, Yes=1
20	Stroke *	No=0, Yes=1
21	High blood pressure *	No=0, Yes=1
22	Hypothyroidism *	No=0, Yes=1
23	Deep-vein thrombosis *	No=0, Yes=1
24	High cholesterol *	No=0, Yes=1
25	Breathing: wheeze in last year	No=0, Yes=1
26	Pneumonia *	No=0, Yes=1
27	Chronic bronchitis/emphysema *	No=0, Yes=1
28	Asthma *	No=0, Yes=1
29	Rheumatoid arthritis *	No=0, Yes=1
30	Osteoarthritis *	No=0, Yes=1
31	Gout *	No=0, Yes=1
32	Osteoporosis *	No=0, Yes=1
33	Hayfever, allergic rhinitis or eczema *	No=0, Yes=1
34	Psoriasis *	No=0, Yes=1
35	Any cancer diagnosis *	No=0, Yes=1
36	Multiple cancers diagnosed	No cancer or single cancer=0, Multiple cancers=1
37	Chest pain	No=0, Yes=1
38	Head and/or neck pain	No=0, Yes=1
39	Back pain	No=0, Yes=1
40	Stomach/abdominal pain	No=0, Yes=1
41	Hip pain	No=0, Yes=1
42	Knee pain	No=0, Yes=1
43	Whole-body pain	No=0, Yes=1
44	Facial pain	No=0, Yes=1
45	Sciatica *	No=0, Yes=1
46	Gastric reflux *	No=0, Yes=1
47	Hiatus hernia *	No=0, Yes=1
48	Gall stones *	No=0, Yes=1
49	Diverticulitis *	No=0, Yes=1

*Self-reported medically diagnosed conditions.

© 2024 Ortolá R et al. *JAMA Network Open.*

eTable 2. Association of wine preference and drinking during meals with mortality in older drinkers from the UK Biobank cohort.

	All-cause mortality		Cancer mortality		CVD mortality	
	n deaths/n	HR (95% CI)[a]	n deaths/n	HR (95% CI)[a]	n deaths/n	HR (95% CI)[a]
**Wine preference and drinking during meals**						
No wine preference nor drinking only during meals	7864/52793	Ref.	3706/52760	Ref.	1699/52760	Ref.
Wine preference or drinking only during meals	4842/45336	0.94 (0.90; 0.97)***	2448/45315	0.94 (0.89; 0.99)*	952/45315	0.94 (0.87; 1.02)
Wine preference and drinking only during meals	3127/36974	0.88 (0.84; 0.92)***	1717/36963	0.91 (0.85; 0.97)**	564/36963	0.89 (0.80; 0.99)*
**Health-related risk factors**						
p-int		0.56		0.72		0.97
No						
No wine preference nor drinking only during meals	2849/25018	Ref.	1519/25004	Ref.	549/25004	Ref.
Wine preference or drinking only during meals	2118/24268	0.95 (0.90; 1.01)	1188/24262	0.96 (0.89; 1.04)	374/24262	0.93 (0.81; 1.06)
Wine preference and drinking only during meals	1500/21139	0.89 (0.84; 0.95)***	887/21130	0.93 (0.85; 1.01)	247/21130	0.87 (0.75; 1.02)
Yes[b]						
No wine preference nor drinking only during meals	5015/27775	Ref.	2187/27756	Ref.	1150/27756	Ref.
Wine preference or drinking only during meals	2724/21068	0.93 (0.88; 0.97)**	1260/21053	0.93 (0.86; 0.99)*	578/21053	0.94 (0.85; 1.05)
Wine preference and drinking only during meals	1627/15835	0.86 (0.81; 0.91)***	830/15833	0.90 (0.82; 0.98)*	317/15833	0.89 (0.78; 1.02)
**By socioeconomic vulnerability**						
p-int		**<0.001**		**0.04**		0.33
No						
No wine preference nor drinking only during meals	4982/37823	Ref.	2449/37797	Ref.	1041/37797	Ref.
Wine preference or drinking only during meals	3711/36235	0.96 (0.92; 1.00)	1905/36217	0.96 (0.90; 1.02)	718/36217	0.96 (0.87; 1.06)
Wine preference and drinking only during meals	2498/29971	0.91 (0.87; 0.96)***	1385/29963	0.94 (0.88; 1.01)	437/29963	0.91 (0.81; 1.02)
Yes[c]						
No wine preference nor drinking only during meals	2882/14970	Ref.	1257/14963	Ref.	658/14963	Ref.
Wine preference or drinking only during meals	1131/9101	0.87 (0.81; 0.93)***	543/9098	0.90 (0.81; 1.00)*	234/9098	0.87 (0.75; 1.01)
Wine preference and drinking only during meals	629/7003	0.73 (0.67; 0.80)***	332/7000	0.79 (0.70; 0.90)***	127/7000	0.79 (0.65; 0.96)*

* p <0.05; ** p <0.01; *** p <0.001. CI = confidence interval; HR = hazard ratio; p-int = p for interaction.

[a] Cox regression model adjusted for sex, age, ethnicity, education, region of the assessment center, smoking status (never, former, or current), physical activity (METs-h/week, tertiles), TV watching time (h/day, tertiles), diabetes, cardiovascular disease, cancer, Townsend deprivation index score (except when stratifying by socioeconomic vulnerability), frailty index score (except when stratifying by health-related vulnerability), and average alcohol intake (g/day, quintiles).

[b] Frailty index score >0.12.

[c] Townsend deprivation index score >0.

© 2024 Ortolá R et al. *JAMA Network Open.*

eTable 3. Association of mean alcohol intake status with mortality in older drinkers from the UK Biobank cohort, excluding participants with prevalent cancer at baseline for cancer mortality, or those with prevalent CVD at baseline for CVD mortality.

	**Cancer mortality**		**CVD mortality**	
	**n deaths/n**	**HR (95% CI)[a]**	**n deaths/n**	**HR (95% CI)[a]**
Occasional drinkers	386/10620	Ref.	183/11070	Ref.
Low-risk drinkers	2332/50194	1.13 (1.01; 1.26)*	941/50933	0.91 (0.77; 1.06)
Moderate-risk drinkers	1898/37212	1.19 (1.06; 1.33)**	649/37987	0.86 (0.72; 1.01)
High-risk drinkers	1543/22635	1.45 (1.29; 1.62)***	571/23086	1.11 (0.93; 1.32)
**Health-related risk factors**				
Overall p-int		0.06		0.13
Low-risk drinkers p-int		0.15		0.80
Moderate-risk drinkers p-int		0.25		0.24
High-risk drinkers p-int		**0.02**		0.86
No				
Occasional drinkers	179/5011	Ref.	70/5405	Ref.
Low-risk drinkers	1181/27693	1.03 (0.88; 1.20)	425/29173	0.88 (0.68; 1.14)
Moderate-risk drinkers	967/20555	1.10 (0.94; 1.29)	327/21718	0.94 (0.73; 1.23)
High-risk drinkers	684/11609	1.24 (1.05; 1.47)*	242/12260	1.12 (0.86; 1.47)
Yes[c]				
Occasional drinkers	207/5609	Ref.	113/5665	Ref.
Low-risk drinkers	1151/22501	1.21 (1.04; 1.40)*	516/21760	0.92 (0.75; 1.13)
Moderate-risk drinkers	931/16657	1.25 (1.08; 1.46)**	322/16269	0.77 (0.62; 0.96)*
High-risk drinkers	859/11026	1.63 (1.40; 1.90)***	329/10826	1.09 (0.87; 1.35)
**Socioeconomic risk factors**				
Overall p-int		0.47		0.21
Low-risk drinkers p-int		0.90		0.46
Moderate-risk drinkers p-int		0.35		0.92
High-risk drinkers p-int		0.47		0.21
No				
Occasional drinkers	269/7716	Ref.	127/8152	Ref.
Low-risk drinkers	1773/39651	1.11 (0.98; 1.27)	688/40515	0.87 (0.71; 1.05)
Moderate-risk drinkers	1377/29121	1.15 (1.00; 1.31)*	481/29945	0.85 (0.70; 1.04)
High-risk drinkers	1024/16438	1.40 (1.22; 1.61)***	351/16946	1.02 (0.83; 1.26)
Yes[c]				
Occasional drinkers	117/2904	Ref.	56/2918	Ref.
Low-risk drinkers	559/10543	1.13 (0.93; 1.38)	253/10418	0.99 (0.74; 1.32)
Moderate-risk drinkers	521/8091	1.28 (1.05; 1.57)*	168/8042	0.84 (0.62; 1.14)
High-risk drinkers	519/6197	1.54 (1.25; 1.88)***	220/6140	1.29 (0.95; 1.73)

* p <0.05; ** p <0.01; *** p <0.001. CI = confidence interval; HR = hazard ratio; p-int = p for interaction.

Occasional drinkers: ≤20 g/week; low-risk drinkers: >20 g/week to ≤ 20g/day for men and >20 g/week to ≤10 g/day for women; moderate-risk drinkers: >20 to ≤40 g/day for men and >10 to ≤20 g/day for women; high-risk drinkers: >40 g/day for men and >20 g/day for women.

[a] Cox regression model adjusted for sex, age, ethnicity, education, region of the assessment center, smoking status (never, former, or current), physical activity (METs-h/week, tertiles), TV watching time (h/day, tertiles), diabetes, cardiovascular disease, cancer, Townsend deprivation index score (except when stratifying by socioeconomic vulnerability), frailty index score (except when stratifying by health-related vulnerability), wine preference and drinking during meals.

[b] Frailty index score >0.12.

[c] Townsend deprivation index score >0.

© 2024 Ortolá R et al. *JAMA Network Open.*

eTable 4. Association of wine preference or drinking during meals with mortality in older drinkers from the UK Biobank cohort, excluding participants with prevalent cancer at baseline for cancer mortality, or those with prevalent CVD at baseline for CVD mortality.

	Cancer mortality		CVD mortality	
	**n deaths/n**	**HR (95% CI)[a]**	**n deaths/n**	**HR (95% CI)[a]**
**Wine preference**				
No wine preference	4366/76112	Ref.	1731/75695	Ref.
Wine preference	1793/44549	0.95 (0.90; 1.02)	613/47381	0.90 (0.81; 1.00)
**Health-related risk factors**				
p-int		0.26		0.71
No				
No wine preference	2027/39130	Ref.	766/40893	Ref.
Wine preference	984/25738	0.98 (0.91; 1.07)	298/27663	0.88 (0.77; 1.02)
Yes[c]				
No wine preference	2339/36982	Ref.	965/34802	Ref.
Wine preference	809/18811	0.92 (0.85; 1.01)	315/19718	0.91 (0.80; 1.05)
**Socioeconomic risk factors**				
p-int		0.40		0.08
No				
No wine preference	3048/57256	Ref.	1170/57524	Ref.
Wine preference	1395/35670	0.97 (0.90; 1.04)	477/38034	0.94 (0.84; 1.06)
Yes[c]				
No wine preference	1318/18856	Ref.	561/18171	Ref.
Wine preference	398/8879	0.91 (0.81; 1.03)	136/9347	0.78 (0.64; 0.95)*
**Drinking during meals**				
No drinking only during meals	3539/59571	Ref.	1343/59264	Ref.
Drinking only during meals	2620/61090	0.93 (0.88; 0.99)*	1001/63812	0.97 (0.89; 1.07)
**Health-related risk factors**				
p-int		0.40		0.50
No				
No drinking only during meals	1590/29744	Ref.	560/31091	Ref.
Drinking only during meals	1421/35124	0.95 (0.88; 1.03)	504/37465	1.00 (0.88; 1.14)
Yes[c]				
No drinking only during meals	1949/29827	Ref.	783/28173	Ref.
Drinking only during meals	1199/25966	0.91 (0.85; 0.99)*	497/26347	0.94 (0.84; 1.06)
**Socioeconomic risk factors**				
p-int		0.13		**0.03**
No				
No drinking only during meals	2363/43373	Ref.	1193/48211	Ref.
Drinking only during meals	2080/49553	0.95 (0.89; 1.01)	1003/55766	1.02 (0.92; 1.13)
Yes[c]				
No drinking only during meals	1176/16198	Ref.	725/18049	Ref.
Drinking only during meals	540/11537	0.87 (0.78; 0.97)**	294/13012	0.83 (0.70; 0.99)*

* p <0.05; ** p <0.01; *** p <0.001. CI = confidence interval; HR = hazard ratio; p-int = p for interaction.

[a] Cox regression model adjusted for sex, age, ethnicity, education, region of the assessment center, smoking status (never, former, or current), physical activity (METs-h/week, tertiles), TV watching time (h/day, tertiles), diabetes, cardiovascular disease, cancer, Townsend deprivation index score (except when stratifying by socioeconomic vulnerability), frailty index score (except when stratifying by health-related vulnerability), average alcohol intake (g/day, quintiles), and the other drinking pattern.

[b] Frailty index score >0.12.

[c] Townsend deprivation index score >0.

© 2024 Ortolá R et al. *JAMA Network Open.*

eTable 5. Association of wine preference and drinking during meals with mortality in older drinkers from the UK Biobank cohort, excluding participants with prevalent cancer at baseline for cancer mortality, or those with prevalent CVD at baseline for CVD mortality.

	Cancer mortality		CVD mortality	
	**n deaths/n**	**HR (95% CI)[a]**	**n deaths/n**	**HR (95% CI)[a]**
**Wine preference and drinking during meals**				
No wine preference nor drinking only during meals	3012/47614	Ref.	1179/46658	Ref.
Wine preference or drinking only during meals	1881/40455	0.93 (0.87; 0.99)*	716/41643	0.93 (0.85; 1.03)
Wine preference and drinking only during meals	1266/32592	0.89 (0.83; 0.96)**	449/34775	0.89 (0.79; 1.00)*
**Health-related risk factors**				
p-int		0.51		0.97
No				
No wine preference nor drinking only during meals	1323/23277	Ref.	491/24169	Ref.
Wine preference or drinking only during meals	971/22320	0.95 (0.87; 1.03)	344/23646	0.94 (0.82; 1.09)
Wine preference and drinking only during meals	717/19271	0.93 (0.84; 1.02)	229/20741	0.89 (0.75; 1.05)
Yes[c]				
No wine preference nor drinking only during meals	1689/24337	Ref.	688/22489	Ref.
Wine preference or drinking only during meals	910/18135	0.91 (0.84; 0.99)*	372/17997	0.92 (0.81; 1.05)
Wine preference and drinking only during meals	549/13321	0.86 (0.78; 0.95)**	220/14034	0.87 (0.74; 1.03)
**Socioeconomic risk factors**				
p-int		0.34		**0.04**
No				
No wine preference nor drinking only during meals	1980/34142	Ref.	744/33877	Ref.
Wine preference or drinking only during meals	1451/32345	0.93 (0.87; 1.00)	540/33409	0.95 (0.85; 1.06)
Wine preference and drinking only during meals	1012/26439	0.91 (0.84; 0.99)*	363/28272	0.94 (0.82; 1.07)
Yes[c]				
No wine preference nor drinking only during meals	1032/13472	Ref.	435/12781	Ref.
Wine preference or drinking only during meals	430/8110	0.91 (0.81; 1.02)	176/8234	0.88 (0.74; 1.06)
Wine preference and drinking only during meals	254/6153	0.81 (0.70; 0.94)**	86/6503	0.67 (0.53; 0.85)**

* p <0.05; ** p <0.01; *** p <0.001. CI = confidence interval; HR = hazard ratio; p-int = p for interaction.

[a] Cox regression model adjusted for sex, age, ethnicity, education, region of the assessment center, smoking status (never, former, or current), physical activity (METs-h/week, tertiles), TV watching time (h/day, tertiles), diabetes, cardiovascular disease, cancer, Townsend deprivation index score (except when stratifying by socioeconomic vulnerability), frailty index score (except when stratifying by health-related vulnerability), and average alcohol intake (g/day, quintiles).

[b] Frailty index score >0.12.

[c] Townsend deprivation index score >0.

© 2024 Ortolá R et al. *JAMA Network Open.*

eTable 6. Association of average alcohol intake status with mortality in older drinkers from the UK Biobank cohort, by drinking patterns, excluding participants with prevalent cancer at baseline for cancer mortality, or those with prevalent CVD at baseline for CVD mortality.

	**Cancer mortality**		**CVD mortality**	
	**n deaths/n**	**HR (95% CI)[a]**	**n deaths/n**	**HR (95% CI)[a]**
Overall p-int		0.08		0.06
Low-risk drinkers p-int		0.94		0.77
Moderate-risk drinkers p-int		0.50		0.77
High-risk drinkers p-int		0.11		0.07
No wine preference nor drinking during meals				
Occasional drinkers	119/2971	Ref.	62/3013	Ref.
Low-risk drinkers	951/17862	1.17 (0.96; 1.41)	409/17516	0.91 (0.69; 1.19)
Moderate-risk drinkers	960/15265	1.30 (1.07; 1.57)**	319/14903	0.81 (0.62; 1.07)
High-risk drinkers	982/11516	1.65 (1.36; 2.00)***	389/11226	1.19 (0.91; 1.56)
Wine preference or drinking during meals				
Occasional drinkers	133/3596	Ref.	58/3719	Ref.
Low-risk drinkers	822/18078	1.13 (0.94; 1.36)	330/18413	0.98 (0.74; 1.30)
Moderate-risk drinkers	572/12334	1.16 (0.96; 1.40)	198/12722	0.94 (0.70; 1.26)
High-risk drinkers	354/6447	1.32 (1.08; 1.61)**	130/6789	1.16 (0.85; 1.58)
Wine preference and drinking during meals				
Occasional drinkers	134/4053	Ref.	63/4338	Ref.
Low-risk drinkers	559/14254	1.11 (0.92; 1.35)	202/15004	0.84 (0.64; 1.12)
Moderate-risk drinkers	366/9613	1.10 (0.91; 1.35)	132/10362	0.89 (0.66; 1.21)
High-risk drinkers	207/4672	1.24 (0.99; 1.54)	52/5071	0.72 (0.50; 1.04)

* p <0.05; ** p <0.01; *** p <0.001. CI = confidence interval; HR = hazard ratio; p-int = p for interaction.

Occasional drinkers: ≤20 g/week; low-risk drinkers: >20 g/week to ≤ 20g/day for men and >20 g/week to ≤10 g/day for women; moderate-risk drinkers: >20 to ≤40 g/day for men and >10 to ≤20 g/day for women; high-risk drinkers: >40 g/day for men and >20 g/day for women.

[a] Cox regression model adjusted for sex, age, ethnicity, education, region of the assessment center, smoking status (never, former, or current), physical activity (METs-h/week, tertiles), TV watching time (h/day, tertiles), diabetes, cardiovascular disease, cancer, Townsend deprivation index score, and frailty index score.

© 2024 Ortolá R et al. *JAMA Network Open.*
