# Supplementary material for: Alcohol Consumption Patterns and Mortality Among Older Adults With Health-Related or Socioeconomic Risk Factors
Source: JAMA Netw Open. 2024 Aug 12;7(8):e2424495. doi: 10.1001/jamanetworkopen.2024.24495 (PMC11320169; doi:10.1001/jamanetworkopen.2024.24495)
Supplement: Supplement 2. — Data Sharing Statement [file jamanetwopen-e2424495-s002.pdf]

Data Sharing Statement

Ortolá. Alcohol Consumption Patterns and Mortality Among Older Adults With Health-Related or Socioeconomic Risk Factors. *JAMA Netw Open*. Published August 12, 2024. doi:10.1001/jamanetworkopen.2024.24495

Data
Data available: No

Additional Information
Explanation for why data not available: Data cannot be shared publicly because of privacy politics. Data are available from the Universidad Autónoma de Madrid Institutional Data Access for researchers who meet the criteria for access to confidential data
